# Comparison of the effect of bacterial stimulation on the global epigenetic landscape and transcription of immune genes in primarily zoophilic members of the *Anopheles gambiae* complex (Diptera: Culicidae)

**DOI:** 10.1016/j.molbiopara.2024.111631

**Published:** 2024-12

**Authors:** Nashrin F. Patel, Blaženka D. Letinić, Leanne Lobb, Jacek Zawada, Dumsani M. Dlamini, Nondumiso Mabaso, Givemore Munhenga, Shüné V. Oliver

**Affiliations:** a Centre for Emerging Zoonotic and Parasitic Diseases, National Institute for Communicable Diseases of the National Health Laboratory Service, Johannesburg, South Africa; b Wits Research Institute for Malaria, Faculty of Health Sciences, University of the Witwatersrand, Johannesburg, South Africa; c Clinical HIV Research Unit, Wits Health Consortium, Johannesburg, South Africa; d South African National Biodiversity Institute (SANBI) National Zoological Gardens, Pretoria, South Africa

**Keywords:** Epigenetics, Methylation, Histones, Antimicrobial peptides, *Anopheles arabiensis*, Insecticide resistance

## Abstract

Members of the *Anopheles gambiae* complex vary in their vector competence, and this is often attributed to behavioural differences. Similarly, there are differences in transmission capabilities of the zoophilic members of this complex despite exhibiting similar behaviours. Therefore, behavioural differences alone cannot fully explain vector competence variation within members of the *An. gambiae* complex. The immune system of mosquitoes plays a key role in determining susceptibility to parasite infection and consequently transmission capacity. This study aimed to examine variations in the immune response of *An. arabiensis*, *An. merus* and *An. quadriannulatus*, a major, minor, and non-vector respectively. The global epigenetic landscape was characterised and the expression of *Defensin-1* and *Gambicin* was assessed in response to Gram-positive (*Streptococcus pyogenes*) and Gram-negative (*Escherichia coli*) bacterial infections. The effect of insecticide resistance in *An. arabiensis* on these aspects was also assessed. The immune system was stimulated by a blood-borne bacterial supplementation. The 5mC, 5hmC, m6A methylation levels and Histone Acetyl Transferase activity were assessed with commercial ELISA kits. The transcript levels of *Defensin-1* and *Gambicin* were assessed by quantitative Real-Time Polymerase Chain Reaction. Species-specific differences in 5mC and m6A methylation existed both constitutively as well as post immune stimulation. The epigenetic patterns observed in the laboratory strains were largely conserved in F1 offspring of wild-caught adults. The methylation patterns in the major vector typically differed from that of the minor/non-vectors. The differences between insecticide susceptible and resistant *An. arabiensis* were more reflected in the expression of *Defensin-1* and *Gambicin*. The expression of these peptides differed in the strains only after bacterial stimulation. *Anopheles merus* and *An. quadriannulatus* expressed significantly higher levels of antimicrobial peptides, both constitutively and after immune stimulation. These findings suggest molecular variations in the immune response of members of the *An*. *gambiae* complex.

## Highlights

• Members of the *An. gambiae* complex differ in epigenetic markers.
• Immune stimulation differentially alters methylation and Histone Acetyl Transferase activity.
• Major vectors have significantly lower Antimicrobial Peptide expression.

## Graphical Abstract

**Figure:**
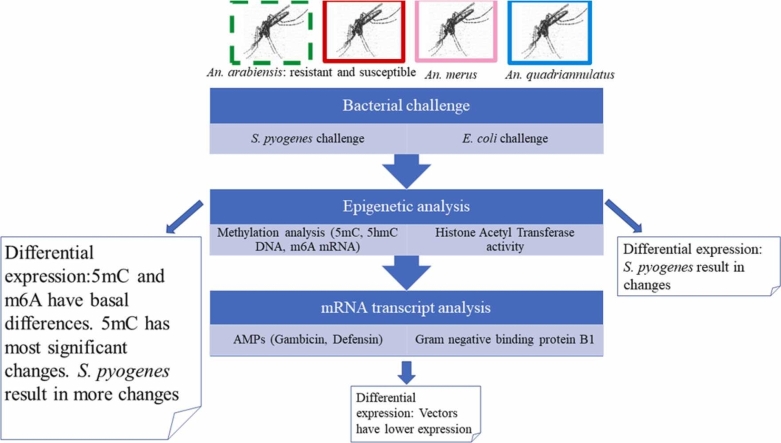

## 1 Introduction

The *Anopheles gambiae* complex consists of nine species that vary in their capability of transmitting the human malaria parasite *Plasmodium falciparum*. These differences in vector competence exist despite their phenotypic similarities and genetic relatedness [1], [2]. In South Africa, three members of this complex are present: *An. arabiensis*, *An. merus* and *An. quadriannulatus*. Of the three members found in South Africa, *An. arabiensis* has been implicated in malaria transmission the most [3] and is considered a dominant vector species in most parts of Africa [4]. *Anopheles merus* is a less efficient vector of malaria, however, under certain situations it has the potential to transmit malaria. It is therefore considered as a minor vector [5], [6]. There are no records of *An. quadriannulatus* transmitting the malaria parasite under natural settings, hence it is considered as a non-vector [7].

The difference in capacity to transmit malaria is referred to as vector competence [8]. Difference in behaviour may underlie the variation in vector competencies exhibited by members of the *An. gambiae* complex. The most efficient vectors in the complex *An. gambiae* and *An. colluzzi* are highly anthropophilic [4]. *Anopheles arabiensis*, which exhibits both exophily and exophagy, is moderately efficient but still considered a major vector [4]. Interestingly, both *An. merus* and *An*. *quadriannulatus* are largely zoophilic but differ in their vector competence. Therefore, behavioural difference alone cannot fully explain the difference in vector competence between members of the *An. gambiae* complex [4], [9], [10]. It could be possible that differences in immune response within this complex may also play a role in variation in vector competence.

The immune system plays a key role in influencing whether *Plasmodium* infection establishes and persists within the *Anopheles* mosquito. The survival and reproduction of *Plasmodium* parasites depends on the efficacy of the mosquito’s immune response [11]. An efficient immune response would reduce the transmission capacity of the mosquito. There are many immunological factors in the humoral and cellular immune responses that contributes to differences in vectorial capacity between species (reviewed by [12]). These factors are found both upstream and or downstream of the immune signalling receptors (Finlay and McFadden, 2006). Pathogen recognition receptors (PRRs) are upstream factors that recognize pathogens and initiate immune signalling pathways [13]. Gram-negative binding protein (GNBP) and Peptidoglycan recognition protein (PGRP) are some of the PRRs that detect fungi, Gram-positive and Gram-negative bacteria in insects [14], [15]. On the other hand, antimicrobial peptides (AMPs) that are synthesised as a result of the activated immune signalling pathway constitute the downstream effector molecules that neutralises infections (reviewed by [16]). To date, four type of AMPs (Defensin, Cecropin, Attacin and Gambicin) have been isolated from different mosquito species. Defensins and Gambicin have been demonstrated to have anti-*Plasmodium* activity, and as such, were examined in this study (reviewed by [17]).

Most examinations of mosquito immunity have occurred in *An. stephensi* [18], [19], [20], [21]*, An. albimanus*[22], [23], [24]*, and An. gambiae* [25], [26]. As such, differences in the immune responses of the minor and non-vectors within the *An. gambiae* complex are poorly explored. There is, however, evidence that the complement system of *An. quadriannulatus* is triggered to respond at lower levels of *Plasmodium* parasite than *An. colluzzi* and *An. arabiensis* [27]. This suggests that differences in the immune response play a significant role in vector competence within the complex.

Another potential molecular mechanism that could underlie species-specific differences in vector competence is variation in epigenetic architecture. Epigenetics is the study of the modulation of gene expression without altering the deoxyribonucleic acid (DNA) sequence. Modulation of epigenetic markers results in rapid, non-germline changes in gene regulation [28]. Alteration of the epigenetic landscape upon immune stimulation could result in a differential immune response through altered gene regulation. Epigenetic variations have been shown to play a role in variations in; cold tolerance [29], insecticide tolerance [30] and responses to *Plasmodium* infection between different mosquito species [31]. For example, parasite infection results in large scale chromatin rearrangements in *Anopheles* mosquitoes [31], indicating the crucial role of epigenetics in the immune response to *Plasmodium* infection. Involvement of all the above shows the complexity of determining susceptibility to parasite infections by mosquitoes. Understanding these factors is critical in the development of novel parasite transmission strategies. The aim of this study was to determine the expression of antimicrobial peptides (AMPs) and to characterise the epigenetic landscape in the zoophilic members of the *An. gambiae* complex, in relation to their relative immune response and if these responses are affected by insecticide resistance. This was done by assessing the expression of *Defensin*-*1* (Def-1) and *Gambicin* (Gamb) and characterising the variation in specific epigenetic markers in *An. arabiensis*, *An. merus* and *An. quadriannulatus* in response to *Streptococcus pyogenes* (Gram-positive) and *Escherichia coli* (Gram-negative) bacterial infections.

## 2 Materials and methods

### 2.1 Mosquito strains

Two strains of *An. arabiensis* (SENN and SENN DDT) and one strain each of *An. merus* (MAFUS) and of *An. quadriannulatus* (SANGWE) were used in this study. SENN is an insecticide susceptible laboratory strain, colonised in 1980 from material collected from Sennar, Sudan. SENN DDT is an insecticide resistant laboratory strain. This strain has been under constant selection with 4 % DDT since 2004. It is fixed for the L1014F *kdr* mutation and displays elevated insecticide detoxification enzyme levels [32]. As such, the strain displays resistance to multiple insecticide classes [33]. MAFUS is an insecticide tolerant (low level pyrethroid resistance) laboratory strain of *An. merus*, colonised in 2012 from Mafayeni, Kruger National Park, South Africa. SANGWE is an insecticide susceptible laboratory strain of *An. quadriannulatus*, colonized in 1998 from Sangwe, Zimbabwe.

The F1 strain of progeny of the wild-caught *An. arabiensis* from Mamfene, Kwazulu-Natal, South Africa, that displayed low levels of bendiocarb, and permethrin resistance was also used. This strain was generated by pooling the progeny of wild collected *An. arabiensis* iso-female lines. The wild specimens were collected between October 2019 and February 2020. This strain was used to represent the wild-type *An. arabiensis* population. All four laboratory strains were housed in the Botha de Meillon insectary located at the National Institute of Communicable Diseases (NICD), Johannesburg, South Africa. Mosquitoes were reared according to the methods of [34]; with a 12:12 hr photoperiod with a 45 min dusk and dawn cycle at 25 °C (±2 °C) and 80 % (±5 %) humidity. Larvae were reared on powdered dog biscuits (Beano™) and Brewer’s yeast mixed at a ratio of 3:1. Adults were maintained with *ad libitum* access to 10 % sucrose.

### 2.2 Immune stimulation by blood-borne bacteria supplementation

Three-day-old female adult mosquitoes from each strain were divided into four equal groups (n=30 mosquitoes per group). Group one received a 10 % sucrose meal only and constituted the untreated or control cohorts. Group two received a single unsupplemented (standard) blood meal. Group three received blood with *S. pyogenes* (ATCC 19615) added. Group four received blood meals with *E. coli* (ATCC 25922) added. The bacterial stock was grown to mid-log phase and the blood was supplemented with bacteria to a final concentration of 0.3 % in 15 ml according to Barnard et al*.*, (2019). The blood treatments took place at the age of 3 days. The blood source was defibrinated cow blood. The meal was provided through an artificial membrane feeder (Hemotek™) for 4 hours. The females were fed to repletion, with successfully fed females separated and were allowed to digest the blood for 72 hours. Only females that were fully fed were used for subsequent experiments. The mosquitoes were then cold-killed and stored at -70℃ until usage.

### 2.3 Calorimetric assessment of global epigenetic markers

DNA, RNA, and nuclear proteins were extracted from each group of each strain in replicates of six with five mosquitoes per replicate. DNA was extracted using a column-based NucleoSpin® DNA insect kit (Macherey-Nagel: PT40101). Samples were mechanically homogenised using a Gene® 2 vortex according to manufacturer’s instructions. The extracted DNA was quantified using a Nanodrop™ 2000 C (Thermo Fisher Scientific) and assessed for purity by TapeStation™ analysis with genomic DNA ScreenTape® (Agilent Technologies). The extracted DNA was utilised in an enzyme-linked immunosorbent assay (ELISA) based on 5-methylcytosine (5mC) and 5-hydroxymethylcytosine (5hmC) quantification, using the Imprint® Methylated DNA Quantification Kit (Sigma-Aldrich®: MDQ1) and the Quest 5-hmC™ DNA ELISA kit (Zymo Research: D5425) respectively, according to manufacturer’s instructions. The absorbance of the final reaction was measured using a SpectraMax® ABS Plus plate reader at 450 nm for 5mC and 405 nm for 5hmC quantification.

RNA was extracted using a column-based Quick-RNA™ MiniPrep kit (Zymo Research: R1054). Samples were mechanically homogenised using a Gene® 2 vortex according to manufacturer’s instructions. Purity and concentration of the extracted RNA were assessed using a Nanodrop™ 2000 C and integrity was confirmed by TapeStation™ analysis with RNA ScreenTape® (Agilent Technologies). RNA integrity was also assessed using 1 % 0.5× Tris-Borate-EDTA (TBE) agarose gel electrophoresis at 70 V, 90 mA for 30 minutes. Samples were sized using a RiboRuler™ High Range RNA Ladder (Thermo Scientific™: SM1821). The extracted RNA was utilized in an ELISA-based N6-methyladenosine (m6A) quantification using the m6A RNA Methylation Assay Kit (Abcam®: ab185912) according to manufacturer’s instructions. The absorbance of the final reaction was measured using SpectraMax® ABS Plus at 450 nm.

For nuclear protein extraction, the bacteria were provided in a 10 % sucrose meal supplemented with either *S. pyogenes* or *E. coli* to represent Gram-positive and Gram-negative immune challenge respectively. This was to prevent any potential blood-borne proteins from affecting the Histone Acetyl Transferase (HAT) activity under immune challenge. Mosquitoes in each sample were homogenised to disrupt the chitinous exoskeleton of mosquitoes in 0.1 M phosphate-buffered saline (PBS) pH 7.0 supplemented with a cocktail of protease inhibitors (2 mM leupeptin, 4 mM PMSF, 0.5 M EDTA). The samples were homogenised with a Tissue Lyser II for 10 min at 25 Hz. The samples were centrifuged (500×g at 4℃) for 2 minutes to precipitate the chitin proteins. The pellet was discarded, and the supernatant was used to extract the nuclear proteins using the NucBuster™ Protein Extraction Kit (Novagen®: 71183). Extracted proteins were quantified using the Bradford assay (Bradford, 1976). A final amount of 50 µg homogenate was used in the Histone acetyltransferase (HAT) Activity Colorimetric Assay kit (Sigma-Aldrich®: EPI001) according to manufacturer’s instructions. The kit is based on the principle of Nicotinamide adenine dinucleotide (NADH) released by the active HAT through acetylation of its substrate histones. NADH was measured calorimetrically at 440 nm on SpectraMax® ABS Plus microplate reader. For all experiments, untreated adults constitute the control group.

### 2.4 Analysis of relative immune-related transcript expression in the *An. gambiae* complex

To examine the effects of immune stimulation on the expression of immune-related genes, messenger RNA (mRNA) transcript levels of three genes were assessed. Immune stimulation was provided by treated sugar water, as per the preparation for HAT activity. Of the three genes, two were AMPs: Defensin *Def-1* (GenBank accession number: AF063402; [35]) and Gambicin *Gamb* (GenBank accession number: AJ237664; [36]). The final transcript was a PRR gene Gram-negative Binding Protein *GNBP-1* (GenBank accession number: AF228472; [37])*.* The three genes were amplified using the primer sequences detailed in Table 1 in a quantitative Real Time Polymerase Chain Reaction (qRT-PCR). The 26 S and 18 S genes were used as reference genes, with the primer sequences detailed in Table 1. The RNA was extracted from the control and immune-stimulated mosquitoes using the Quick-RNA™ MiniPrep kit as detailed in Section 2.3. The RNA was used to generate 1000 nM of complementary DNA (cDNA) in replicates of three, using the iScript™ cDNA Synthesis Kit (Bio-Rad: 1708891) according to manufacturer’s instructions. The cDNA (100 nM) was used as a template for the qRT-PCR reaction in SsoAdvanced™ Universal SYBR® Green Supermix (Bio-Rad: 1725270) according to manufacturer’s instructions with the following cycling conditions: DNA denaturation at 98 °C (3 min) followed by 40 cycles at 95 °C (10 sec) with a final extension at 60 °C (30 sec). For all experiments, untreated adults constitute the control group.

**Table 1:** Primer sequences of the target and reference genes.

**Primer name**	**Primer sequence**
*Defensin-1* forward primer	5′ GGA CAA CTA GGA AGG ACA AAC A 3′
*Defensin-1* reverse primer	5′’ ACG GTA GAG TCC TGA GGT AAA 3′
*Gambicin* forward primer	5′ TTT GCT GCT CGG TTG AGT 3′
*Gambicin* reverse primer	5′ TGC AGT CCT CAC AGC TAT TG 3′
*Gram-negative binding protein-1* forward primer	5′ GGA CCA GTT TGG GCT AGA TT 3′
*Gram-negative binding protein-1* reverse primer	5′ATT CCC GAT TCG AAG GTT ATC A 3′
*18S* forward primer (Reference gene)	5′ TAC CTG GGC GTT CTA CTC 3′
*18S* reverse primer (Reference gene)	5′ CTT TGA GCA CTC TAA TTT GTT C 3′
*26S* forward primer (Reference gene)	5′ GAT AAG GCA ATC AAG AAG TTC G 3′
*26S* reverse primer (Reference gene)	5′ TAC GGA CAA CCT TCG AGT GG 3′

### 2.5 Statistical analysis

The statistical packages Statistix 8™ (Analytical Software, Tallahassee, Florida) and SPSS v22 (IBM Corp. Released 2013. IBM SPSS Statistics for Windows, Version 22.0. Armonk, NY: IBM Corp.) were used for analysis. The Shapiro-Wilk test was performed to determine normality [38]. Non-parametric data were assessed using a Kruskal-Wallis Analysis of Variance (ANOVA) [39]. Parametric data were assessed using One-Way ANOVA [38]. Tukey’s honest significant (HSD) test was used post One-way ANOVA [40] and Kruskal-Wallis All-Pairwise Comparisons was used post Kruskal-Wallis ANOVA [39]. Differences in transcripts levels were assessed by the Pfaffl method [41] by One-Way ANOVA using Bio-Rad CFX maestro™ software. An alpha value of 0.05 was applied to all analysis.

### 2.6 RESULTS

#### 2.6.1 Characterisation of 5mC within laboratory strains

**Fig. 1:**
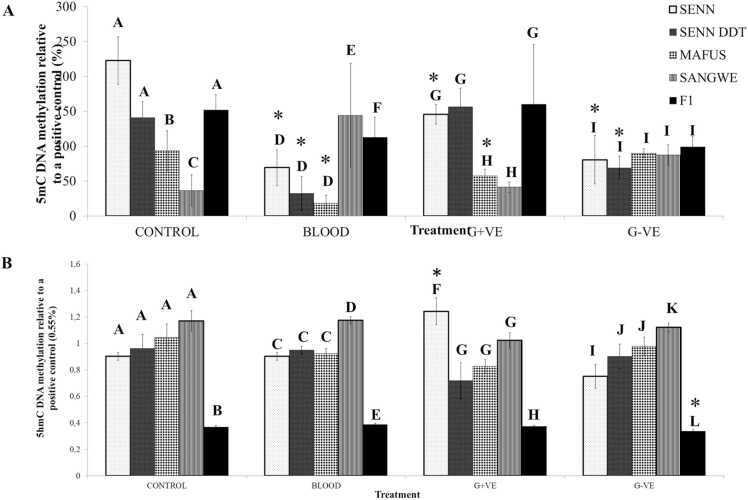
Global changes in DNA methylation levels in laboratory strains and F1 *An. arabiensis* in response to immune stimulation administered via a blood meal. Calorimetric analysis of DNA methylation levels compared to an internal positive control determined by Enzyme Linked Immunosorbent Assay (ELISA) (A) Changes in 5-methylcysteine (5mC) levels. Data is presented as relative % methylation. (B) Changes in 5-hydroxymethyl cysteine (5hmC) levels. Data is presented as relative to a 0.55 % methylated DNA control. SENN–insecticide susceptible *An. arabiensis*, SENN DDT–insecticide resistant *An*. *arabiensis*, MAFUS–*An. merus,* SANGWE–*An. quadriannalatus* and F1–Progeny of wild *An. arabiensis*. Asterisks (*) indicate a significant difference from the control of the same strain. Significant differences between strains within the same treatment are indicated by capital letters.

The 5mC methylation was generally more methylated than the kit-provided positive control. Blood treatment and Gram-positive treatment had the greatest effect on the 5-mC levels, and the changes were typically a decrease from the control level (Fig. 1A).

There was a significant difference in the 5mC methylation levels of the four laboratory strains (One-Way ANOVA: p < 0.01, F _(3, 20)_ = 8.22). Pairwise comparison demonstrated that the major vector strains (SENN & SENN DDT) had significantly higher levels of 5mC methylation levels than that of MAFUS and SANGWE. The 5mC levels between SENN and SENN DDT were not significantly different (p = 0.07, F _(1, 11_) = 4.00). The 5mC levels in SANGWE were significantly lower than that of MAFUS (p < 0.01, F _(1, 11)_ = 6.89).

When provided with an untreated blood meal resulted in a change in 5mC methylation levels compared to their untreated controls (Kruskal-Wallis ANOVA: p = 0.01; F _(3, 23)_ = 4.84; χ^2^=9.64). Pairwise comparison demonstrated that 5mC methylation in SANGWE was significantly higher than the other three strains. When comparing whether untreated blood resulted in a significant change in 5mC methylation compared to their untreated controls there was a significant decrease for all but SANGWE (*An. quadriannulatus*) and the F1 *An. arabiensis* (p < 0.01; F _(7, 47)_ = 7.25; χ^2^=26.28). Pairwise comparison demonstrated than SENN, SENN-DDT, MAFUS but not SANGWE had a significant reduction in 5mC levels after blood feeding.

Gram positive exposure resulted in a significant change in 5mC levels between the strains (One-Way ANOVA: p < 0.01, F _(3, 20)_ = 13.4). Pairwise comparison demonstrated that SENN and SENN-DDT did not differ from each other, and MAFUS and SANGWE did not differ from each other. SENN and SENN-DDT 5mC levels post Gram-positive treatment were, however, significantly higher than that of MAFUS and SANGWE. When comparing 5mC levels after Gram-positive treatment compared to their control counterparts, there was a significant difference (p < 0.01, F _(7, 47)_ = 8.48). Pairwise comparison demonstrated that SENN and MAFUS had significantly lower 5mC levels after Gram-positive treatment compared to their untreated controls.

Gram-negative treatment did not result in difference in 5mC levels between the four strains (One-Way ANOVA: p = 0.89, F _(3, 23)_ = 0.20). When comparing the effect of Gram-negative treatment on the strains compared to their control counterparts, there was a significant change (p < 0.01, F _(7, 47)_ = 5.37). Pairwise comparison demonstrated that SENN and SENN-DDT had a significant reduction in 5mC levels post Gram-negative treatment compared to their untreated counterparts.

#### 2.6.2 Characterisation of 5hmC levels within laboratory strains

By contrast to 5mC levels, 5hmC levels were remarkably unchanged by treatment. The differences between the strains were not marked, with SANGWE typically having the highest level of 5hmC methylation (Fig. 1B).

There was no significant difference in 5hmC levels between the four laboratory strains of the control group (One-Way ANOVA: p = 0.24, F _(3, 19)_ = 1.56). Blood treatment resulted in a significant difference in 5hmC methylation levels between the strains (p < 0.01, F _(4, 21)_ = 99.5). Pairwise comparison revealed that there was a significant increase in 5hmC levels in SANGWE post blood ingestion in comparison to SENN, SENN DDT and MAFUS. There was no significant difference in the 5hmC levels between the blood treated strains and the untreated counterparts four untreated laboratory strains and strain treated with blood (p = 0.03, F _(7, 39)_ = 2.61. A Tukey post-hoc test showed that the results were not significant: Q=4.58, critical value=0.321).

Exposure to Gram-positive bacteria resulted in a significant difference in 5hmC levels in between the strains (One-Way ANOVA: p < 0.01, F _(3, 23)_ = 6.14). Subsequent pairwise comparison demonstrated that the significant difference was an increase in 5hmC levels in SENN compared to SENN DDT, MAFUS and SANGWE in comparison to SENN. When comparing 5hmC levels after Gram-positive treatment to their respective untreated controls there was a significant difference (p < 0.01, F _(7, 43)_ = 3.60). Pairwise comparison indicated that there was a significant increase in 5hmC levels in SENN after Gram-positive treatment.

Statistical analysis showed a significant difference in in 5hmC levels after exposure to Gram- negative bacteria between the 4 cohorts (One-Way ANOVA: p = 0.02, F _(3, 23)_ = 4.24). Subsequent pairwise comparisons showed that exposure to Gram-negative bacteria resulted in in SANGWE having the highest 5hmC levels, SENN having the lowest, with SENN-DDT and MAFUS not differing from each other. Comparison of the effect of Gram-negative bacteria on 5hmC levels compared to control levels showed a suggested a significant change after Gram-negative treatment (p = 0.02, F _(7, 43)_ = 2.65). Subsequent pairwise comparison demonstrated that none of the treatments differed significantly from their untreated controls (Tukey’s post hoc test: critical Q value = 4.55).

#### 2.6.3 Characterisation of m6A mRNA methylation within laboratory strains

**Fig. 2:**
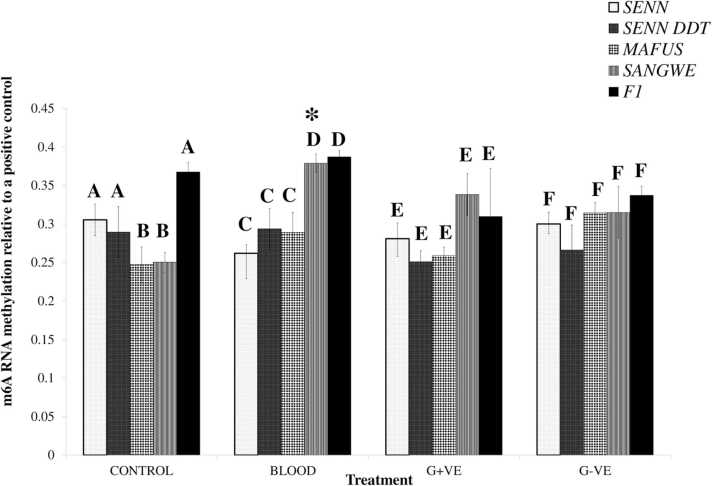
Global changes in N6-methyladenosine m6A mRNA methylation levels in laboratory strains and F1 *An. arabiensis* in response to immune stimulation administered via a blood meal. Calorimetric analysis of m6A RNA methylation levels compared to an internal positive control determined by Enzyme Linked Immunosorbent Assay (ELISA). Asterisks (*) indicate a significant difference from the control of the same strain. Significant differences between strains within the same treatment are indicated by capital letters.

Although species-specific differences in m6A mRNA methylation existed, the treatments typically did not induce a change in methylation levels compared to the untreated controls. The most notable finding is that methylation levels increased in the non-vector SANGWE regardless of treatment (Fig. 2).

The m6A mRNA methylation levels of the strains were significantly different under control conditions (One-Way ANOVA: p = 0.01, F _(3, 23)_ = 5.23). Pairwise comparison showed that m6A mRNA methylation levels of major vectors (SENN & SENN DDT) were significantly higher than the minor/non-vectors (MAFUS & SANGWE). The m6A levels of SENN and SENN DDT were not significantly different under control condition. This was also true for MAFUS and SANGWE.

No significant differences in the m6A levels were observed upon blood treatment in SENN, SENN DDT and MAFUS (One-Way ANOVA: p = 0.55, F _(5, 35)_ = 0.810). However, increased m6A levels were observed in SANGWE compared to the untreated control (p < 0.01, F _(3, 20)_ = 5.23). A significant increase in m6A levels was also observed in SANGWE in comparison to SENN, SENN DDT and MAFUS post blood ingestion (p < 0.01, F _(4, 25)_ = 9.42).

After blood treatment there was a significant difference in m6A mRNA methylation levels between the strains (One-Way ANOVA: p < 0.01, F _(3, 23)_ = 6.20). Subsequent pairwise comparisons indicated that SANGWE had significantly higher m6A mRNA methylation levels than the other three stains. When comparing m6A methylation levels after blood treatments compared their untreated controls, there was a significant difference (One-Way ANOVA: p < 0.01, F _(7, 47)_ = 3.65). Pairwise comparison showed that only SANGWE had a significant increase in m6A mRNA methylation after blood feeding.

Gram-positive exposure resulted in a significant difference in m6A mRNA methylation between the strains (One-Way ANOVA: p = 0.02, F _(3, 23)_ = 4.13). This significant difference was due to a significantly higher level of m6A methylation levels in SANGWE. When comparing m6A methylation levels after Gram-positive exposure compared to their untreated controls there was a significant difference (p = 0.04, F _(7, 47)_ = 2.26). A Tukey post-hoc test, however, indicated that there were no significant differences.

There was no significant difference in m6A mRNA methylation levels after Gram-negative treatment between the strains (p = 0.51, F _(3, 23)_ = 0.80). Similarly, there was no significant differences in m6A methylation levels compared to their untreated controls (p < 0.01, F _(7,47)_ = 1.28). A Tukey post hoc test as well as Bonferroni correction demonstrated that there were no true significant differences after treatment.

#### 2.6.4 Characterisation of Histone Acetyl Transferase (HAT) activity within laboratory strains

There was no significant difference in the HAT activity between the four laboratory strains of the control group (One-Way ANOVA: p = 0.09, F _(3, 23)_ = 2.47). Untreated blood exposure resulted in a significant difference in HAT activity between the strain (p < 0.01, F _(3, 23)_ = 6.88). Subsequent pairwise comparison indicated that SENN had significantly lower HAT activity after ingestion of untreated blood. When comparing HAT activity after blood treatment compared to their control counterparts, there was a significant difference (p < 0.01, F _(7, 47)_ = 8.64). Pairwise comparison revealed that with the exception of MAFUS, blood treatment resulted in a significant decrease in HAT activity.

Gram-positive treatment resulted in a significant change in HAT activity between the strains (One-Way ANOVA: p < 0.01, F _(3, 23)_ = 8.72). Pairwise comparison demonstrated that SENN-DDT had a significantly higher HAT activity than the other four strains. HAT activity in MAFUS and SANGWE was significantly lower than that of SENN and SENN-DDT post Gram-positive bacterial exposure. When comparing Gram-positive treatment to the control counterparts, there was also a significant difference (p < 0.01, F _(7, 47)_ = 5.69). Pairwise comparison revealed that SENN-DDT had significantly increased HAT activity after Gram-positive treatment.

**Fig. 3:**
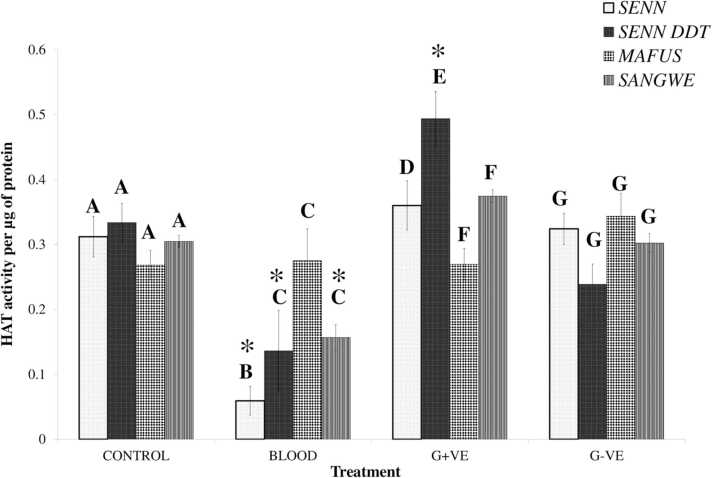
Global changes in Histone Acetyl Transferase (HAT) activity in laboratory strains in response to immune stimulation administered via a sugar-meal. Calorimetric analysis of HAT activity compared to an internal positive control. Asterisks (*) indicate a significant difference from the control of the same strain. Significant differences between strains within the same treatment are indicated by capital letters.

There was no significant difference in HAT activity between the four strains post exposure to Gram-negative bacteria (One-Way ANOVA: p = 0.05, F _(3, 23)_ = 3.17). When comparing HAT activity post Gram-negative treatment to that of untreated controls, there was a significant difference (p = 0.02, F _(3, 23)_ = 8.72). A Tukey post-hoc test revealed that there were no significant differences between Gram-positive treatment and untreated controls (critical Q-value: 4.53; critical value for comparison: 0.12) (Fig. 3).

#### 2.6.5 Comparison of the epigenetic markers of laboratory *An. arabiensis* and F1 progeny of wild *An. arabiensis*

Methylation markers (5mC, 5hmC, and m6A) of the two laboratory strains of *An. arabiensis* (SENN & SENN DDT) were compared to that of F1 progeny of wild-caught *An. arabiensis* at basal levels and post immune stimulation. With the exception of 5hmC levels, there was a remarkable conservation of methylation levels between the laboratory-reared *An. arabiensis* and the progeny of F1 females.

The basal 5mC methylation levels did not differ between the strains (One-Way ANOVA: p = 0.10, F _(2,17)_ = 2.75). This was also true for m6A levels (p = 0.08, F _(2, 17)_ = 3.08). By contrast, the basal 5hmC levels in F1 were significantly lower than the basal 5hmC levels in SENN and SENN DDT (p < 0.01, F _(4, 21)_ = 16.4).

Blood treatment resulted in a significantly higher levels of 5mC (Kruskal-Wallis ANOVA: p < 0.01, F _(4, 25)_ = 5.55, χ^2^ = 13.6) and m6A methylation (One-Way ANOVA: p < 0.01, F _(4, 25)_ = 9.42) in the F1 mosquitoes in comparison to SENN and SENN DDT. By contrast, 5hmC levels were significantly lower in the F1 mosquitoes compared to SENN and SENN DDT post blood treatment (p < 0.01, F _(4, 21)_ = 99.5).

Exposure to Gram-positive bacteria did not result in any significant difference in 5mC levels between SENN, SENN DDT and F1 (Kruskal-Wallis ANOVA: p = 0.17, F _(2, 17)_ = 1.95, χ^2^ = 1.95). This was also true for m6A methylation levels (One-Way ANOVA: p = 0.33, F _(4, 25)_ = 1.22) between the three strains. However, like the blood treatment, 5hmC levels in F1 were significantly lower than the 5hmC levels in SENN and SENN DDT post Gram-positive bacterial exposure (p < 0.01, F _(4, 24)_ = 13.5).

Like Gram-positive bacterial exposure, there was no significant difference in 5mC levels (One-Way ANOVA: p = 0.87, F _(4, 25)_ = 0.310) and m6A levels (p = 0.32, F _(4, 25)_ = 1.25) between SENN, SENN DDT and F1 post Gram-negative bacterial exposure. Gram-negative bacteria also resulted in a significantly lower levels of 5hmC in F1 mosquitoes compared to SENN and SENN DDT (p < 0.01, F _(4, 25)_ = 19.8).

#### 2.6.6 The effects blood treatment and immune stimulation on the epigenetic markers in F1 progeny of wild-caught *An. arabiensis*

Unlike the laboratory strains, the methylation markers F1 strains were not particularly responsive to blood treatment or bacterial challenge. The noticeable exception was a change in 5hmC methylation in response to Gram-negative treatment.

Neither untreated blood nor the bacterial exposure resulted in a significant change in 5mC levels in the F1 progeny in comparison to the untreated F1 controls (One-Way ANOVA: p = 0.76, F _(3, 20)_ = 0.40). Like 5mC, the m6A levels of blood treated and immune stimulated F1 progeny were not significantly different from the untreated F1 controls (p = 0.38, F _(3.23)_ = 1.09). The 5hmC levels in F1 progeny after treatment was significantly different after treatment (p = 0.02, F _(3,22)_ = 4.24). Subsequent pairwise comparison demonstrated that Gram-negative treatment resulted in significantly reduced 5hmC levels.

### 2.7 The effect of immune stimulation on differential Def-1 and Gamb transcripts in the *An. gambiae* complex

#### 2.7.1 *Def-1* and *Gamb* have higher basal expression in MAFUS

**Fig. 4:**
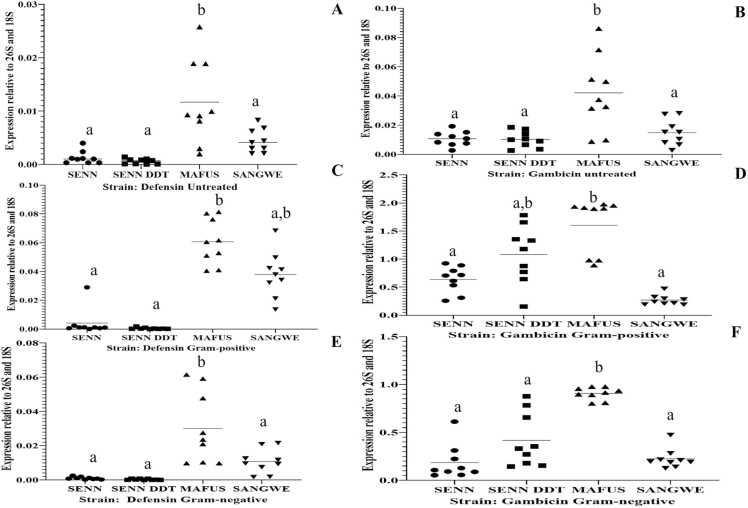
Relative normalized mRNA transcript levels of basal *Defensin-1* and *Gambicin* in laboratory strains. (A) Relative *Defensin-1* (*Def*) expression levels in untreated adults. (B) Relative *Gambicin* (*Gamb*) expression levels in untreated adults. (C) Relative *Defensin-1* (*Def*) expression levels after Gram-positive exposure. (D) Relative *Gambicin* (*Gamb*) expression levels after Gram-positive exposure. (E) Relative *Defensin-1* (*Def*) expression levels after Gram-negative exposure. (F) Relative *Gambicin* (*Gamb*) expression levels after Gram-negative exposure. Significant differences are indicated by different lower-case letter.

At the constitutive level, there were species-specific differences in transcript levels. Basal *Def-1* transcript levels differed y between strains. *Def-1* transcript levels were significantly higher in MAFUS than SENN, SENN DDT and SANGWE (One-Way ANOVA, p<0.01, F_(3, 31)_=10.4). A Tukey HSD post hoc test demonstrated that there was no significant difference between SENN, SENN DDT and SANGWE (Fig. 4A).

**Fig. 5:**
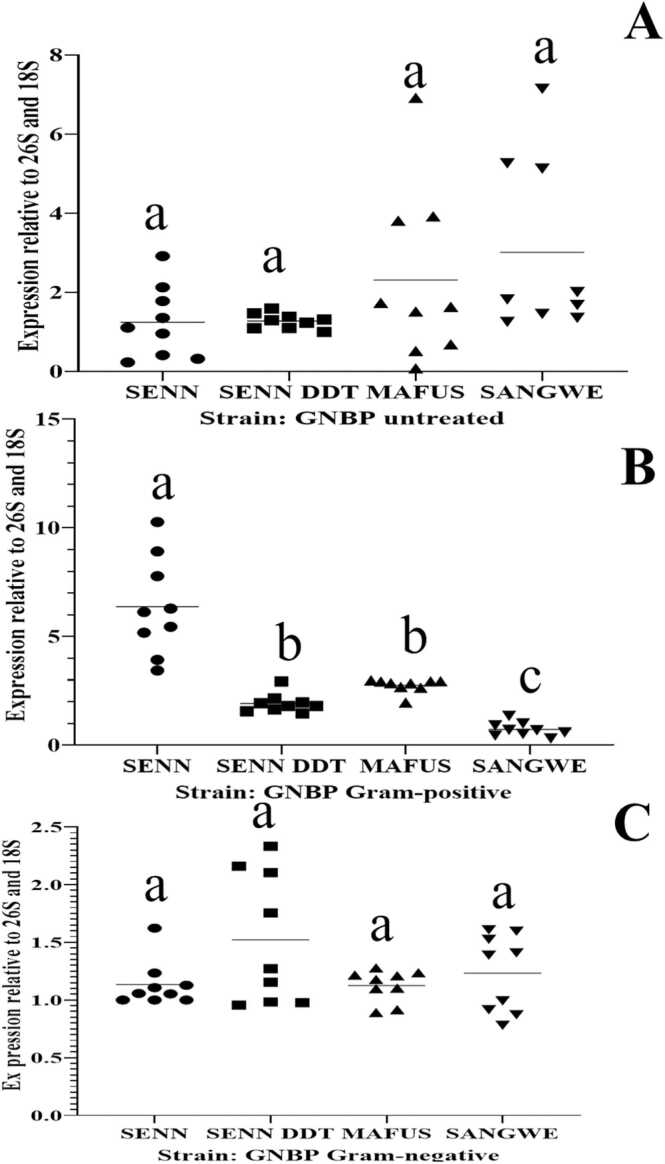
Relative normalized mRNA transcript levels of basal Gram-negative binding protein-1 (*GNBP-1*) and post immune stimulation in laboratory strains. (A) Relative expression levels in untreated adults. (B) Relative expression levels post Gram-positive bacterial challenge. (C) Relative expression levels post Gram-negative. Significant differences are indicated by different lower-case letter.

Like *Def-1*, basal *Gamb* transcript levels were also significantly higher in MAFUS than SENN, SENN DDT and SANGWE (p<0.01, F_(3, 56)_=40.3). A Tukey post-hoc test demonstrated that there was no significant difference between SENN, SENN DDT and SANGWE. *Gamb* transcript levels in SENN DDT were not significantly different from that of SENN (p=0.38, F_(3, 32)_=0.32) (Fig. 4B). There was no constitutive difference in *GNBP-1* transcripts between SENN, SENN DDT, MAFUS and SANGWE (p=0.173, F_(3, 32)_=1.78) (Fig. 5A)**.**

#### 2.7.2 The effects of Gram-positive bacteria exposure on *Def-1* and *Gamb* transcript levels

The species-specific differences observed at the basal level were also observed after Gram-positive treatment. After Gram-positive bacterial exposure *Def-1* transcript levels in MAFUS was significantly higher than SENN and SENN-DDT and (One-Way ANOVA, p < 0.01, F_(3, 21)_=7.30). A Tukey post-hoc test demonstrated that SANGWE did not differ from MAFUS. SENN and SENN-DDT did not differ from each other (Fig. 4C).

*Gamb* transcript levels in MAFUS were significantly higher than that of SANGWE and SENN but not SENN DDT after Gram-positive treatment (p<0.01, F_(1, 19)_=8.9). A Tukey HSD post-hoc test demonstrated that MAFUS and SENN DDT did not differ from each other. SENN DDT was significantly higher than SENN. SENN and SANGWE were not significantly different from each other (Fig. 4D).

Gram-positive treatment was the only treatment that resulted in significant changes in *GNBP-1* between the strains. *GNBP-1* transcript levels in SENN were higher than in SANGWE, SENN and MAFUS post Gram-positive bacterial exposure (p<0.01, F_(3, 32)_=56.73). A Tukey HSD post hoc test demonstrated that MAFUS and SENN-DDT did not differ significantly from each other (Fig. 5B).

#### 2.7.3 The effects of Gram-negative bacterial exposure on *Def-1* and *Gamb* transcript levels

Like with Gram-positive treatment, Gram-negative treatment also resulted in species-specific differences in transcript expression. Upon exposure to Gram-negative bacteria, *Def-1* transcript levels in MAFUS was significantly higher than SENN, SENN-DDT and SANGWE (One-Way ANOVA, p<0.01, F_(3, 43)_=30.8). A Tukey HSD post hoc test demonstrated that SANGWE, SENN and SENN DDT did not differ from each other (Fig. 4E).

*Gamb* transcript levels in MAFUS was significantly higher than in SENN, SENN DDT and SANGWE (p < 0.01, F_(3, 28)_=10.0) (Fig. 4E). A Tukey HSD post hoc test demonstrated that SANGWE, SENN and SENN DDT did not differ from each other. *GNBP-1* transcript levels were not different between strains post Gram-negative bacterial exposure (p=0.09 F_(3, 30)_=0.956**)** (Fig. 5C).

## 3 Discussion

Approximately 70 of the 465 formally recognised *Anopheles* species are known to transmit malaria [4]. The low number of species capable of transmission is an indication of the complex nature of vector competence. There are many factors that determines vector competence in mosquitoes. This study aimed to determine whether zoophilic members of the *An. gambiae* complex, that differ in vector competence, differ in epigenetic landscape and immunological responsiveness.

Data from this study showed that the constitutive levels of 5mC DNA methylation and m6A mRNA methylation in the *An. arabiensis* strains (SENN and SENN DDT) and the *An. merus* (MAFUS) a minor vector and the *An. quadriannulatus* (SANGWE) strains differed significantly. This suggests that even at the constitutive level, there is a difference in the epigenetic make-up of these closely related sibling species. Furthermore, there were constitutive differences in 5mC DNA methylation levels between the minor vector, MAFUS, and the non-vector SANGWE and the two *An. arabiensis* strains. The 5mC methylation levels in the minor and non-vector were lower than that of the two *An. arabiensis* strains. The observed 5mC patterns suggest a lower degree of gene silencing in the minor and non-vector species at the basal level because 5mC DNA methylation generally corresponds to gene silencing [42]. Both 5mC and m6A methylation patterns were conserved in F1 *An. arabiensis* progeny, suggesting that these observations are not a colonisation effect and may be representative of the conditions in the wild.

The effect of m6A mRNA methylation on gene expression as recorded in this study is difficult to interpret. The association with gene silencing or gene expression is dependent on where the methylation occurs [43]. Unfortunately, this cannot be determined by the current experimental methodology. This would require a specific sequence analysis as opposed to the general calorimetric analysis performed in this study. Furthermore, as this study examined m6A mRNA methylation only, it does not exclude that regulatory differences may exist within other forms of RNA.

Blood feeding is known to induce large scale changes in gene expression in mosquitoes [44]. Previous studies have demonstrated both marked changes in over expression and under expression of numerous genes in *An. gambiae* after blood feeding [45]. In general, this study demonstrated that both 5mC DNA methylation and HAT activity was decreased post blood feeding. These changes correspond to decreased gene silencing and decreased gene expression, respectively [42], [46]. This could suggest that reduced 5mC methylation and reduced HAT activity could be related to the regulation of increased and decreased expression of genes in response to blood feeding.

The decrease in 5mC DNA methylation may correspond to observed overexpression of genes involved in defence against reactive oxygen species, blood digesting proteins and detoxification enzymes. The decreased HAT activity may correspond with decreased expression of genes involved in locomotion and response to environmental stimuli [45]. Blood feeding has previously been demonstrated to significantly increase the DNA methyltransferase enzyme *dnmt2* expression in *An. albimanus* [23]. This would have been expected to increase the prevalence of 5mC methylation, but this was generally not observed in this study. This may be due to innate species differences. However, a recent study suggested that for *An. gambiae*, other mechanisms outside of methylation may underlie epigenetic regulation in this species [47]. The results of this study seems to suggest this may be true in the other members of this complex examined in the study.

The exposure to Gram-positive bacteria resulted in a marked difference in epigenetic architecture between the different mosquito species compared to Gram-negative bacterial exposure. *Streptococcus pyogenes* exposure resulted in interspecies differences in 5mC and 5hmC DNA methylation, as well as HAT activity. By contrast, no changes were observed in m6A mRNA methylation levels between species after Gram-positive exposure. Therefore, unless *S. pyogenes* exposure alters m6A methylation levels in other forms of RNA, such tRNA or rRNA, the response to Gram-positive bacteria may be limited to DNA epigenetic modifications.

Levels of 5mC DNA methylation were higher in the major vectors SENN and SENN DDT, which would typically correspond to a greater degree of gene silencing in these species post *S. pyogenes* exposure. This contrasted with higher levels of HAT activity in SENN and SENN DDT strains after a challenge with *S. pyogenes*, which would typically correspond with an increase in transcriptional activity [46]. This could suggest that epigenetic regulation of the transcription response to Gram-positive bacteria may take place at different levels in the major and minor vectors. Although these findings speak to yet unexplored levels of complexity in the regulatory mechanisms, it does suggest a pattern of differential responses to immune stimuli in major and minor/non-vectors.

The interspecies response to Gram-negative bacteria is largely conserved, with no interspecies differences at the 5mC DNA and m6A mRNA methylation, as well as HAT activity levels post *E. coli* exposure. This lack of epigenetic responsiveness to Gram-negative bacteria may be related to the nature of the mosquito’s commensal bacteria. *Anopheles* mosquitoes generally have gut microbiota dominated by Gram-negative bacteria [48]. An epigenetic landscape that is not highly inducible by Gram-negative bacteria may be required to maintain the commensal bacterial population.

Although 5hmC DNA methylation patterns generally did not display notable interspecies differences, there is an exception for Gram-negative bacteria. Post *E. coli* exposure, the insecticide-susceptible major vector SENN had significantly lower levels of 5hmC DNA methylation, which typically correspond with decreased levels of gene expression [49], than SENN DDT and MAFUS, a major vector and a minor vector, respectively. Both SENN DDT and MAFUS display insecticide tolerance, and this may underlie the similar 5hmC epigenetic landscape in response to immune challenge. The 5hmC methylation levels post Gram-negative challenge of all strains was lower than that of the insecticide-susceptible, non-vector, SANGWE. Therefore, although Gram-negative bacteria exposure generally did not induce epigenetic changes, where it does occur, it appears to be regulated by 5hmC DNA methylation alterations.

Basal epigenetic markers were conserved between laboratory strains and F1 progeny at the 5mC and m6A methylation levels. This was also true for the immune stimulation by both types of bacteria. The marked exception was 5hmC levels. This suggests two things. Firstly, that the assays performed on the laboratory strains seems to represent a reasonable approximation of the epigenetic architecture of *An. arabiensis* in the wild. The study on the *An. arabiensis* F1 offspring represents the best approximation of epigenetic architecture possible, as it is difficult to account for the age, number of blood meals and the immunological status if wild caught mosquitoes were to be used directly. Secondly, the low 5hmC DNA methylation levels in the F1 offspring may support previous observation in immunological studies. Previous research has demonstrated a marked exaggeration in the immune response and transcriptome divergence of laboratory-reared mosquitoes compared to their wild counterparts [50], [51]. It has been suggested that this may be due to the relatively unchallenging environment of the laboratory which would allow for greater resource allocation to immunity that would not occur in the wild [52]. In the wild, these resources may be allocated to other physiological functions, such as reproduction. As 5hmC DNA methylation tends to correspond with gene expression [49], the low methylation levels seen in F1 offspring may be related to this hypothesis, with F1 not yet having shifted their resource allocation to immunity. As such, regulation in these mosquitoes may be more related to changes in gene silencing rather than changes in gene activation.

The alteration of the epigenetic landscape in response to immunological challenge has been demonstrated in *An. gambiae*. There is evidence for large scale chromatin rearrangements in *An. gambiae* in response to *P. falciparum* infection [31]. As such, there is a precedent for epigenetic alteration in this species complex as a response to immunological challenge. It is worth noting that the changes are chromatin rearrangements which would be as a result of histone modifications. HAT activity is the most variable after immune challenge in these species. Therefore, epigenetic variation between major vectors, minor vectors and non-vectors may be one of the molecular rather than behavioural factors that alter the capacity to modulate the immune system. This further suggests that these findings, although observed on a global scale, may underlie a difference in immunological responses in the members of the *An. gambiae* complex, and as such, potentially vector competence.

The study design allowed the comparison of epigenetic architecture in different closely related species. The inclusion of an insecticide resistant and susceptible strain from the same genetic background allowed the examination of the role of insecticide resistance on the dynamics of immune stimulation on the global epigenetic landscape. The two strains used (SENN and SENN DDT) were previously demonstrated to display differential epigenetic responses to larval pollutant exposure [53]. This raised the question of whether this would also be reflected in the immune response. Both strains did not differ at the 5mC and m6A methylation levels post both Gram-positive and Gram-negative bacterial treatments, as well as in the HAT activity levels post Gram-negative bacterial exposure. The marked difference observed was the increased level of 5hmC DNA methylation levels and HAT activity in SENN and SENN DDT post Gram-positive bacterial exposure. This indicates a potentially complex regulatory relationships and this is also observed when examining AMPs transcript levels. This may be due to different epigenetic regulatory mechanisms being involved with different aspects of gene expression. Methylation and histone modification may be involved with different aspects of immune regulation. However, the marked changes seen in HAT activity is a repeat of the histone responsiveness to metal stress [54]. This again highlights a previous finding that methylation may not be the primary epigenetic mechanism in *An. gambiae* [47] (and possibly by extension by the rest of the complex), and that histone modification may play a far more significant role in epigenetic regulation in this species complex.

There were no constitutive differences in any of the transcripts examined in this study between the SENN and SENN DDT strains. The inducibility of the transcripts, however, did vary. Gram-positive bacterial exposure reduced *Def-1* and *Gamb* expression in SENN DDT relative to SENN. Similarly, Gram-negative bacterial exposure reduced *Def-1* expression in SENN DDT, however, this treatment increased *Gamb* expression in SENN DDT relative to SENN. This suggests that the insecticide resistance phenotype does not affect the basal immune response, but rather affects the way immune challenge induces the response. The variation in induction patterns varies as much as the epigenetic response, suggesting that some of the immune responses observed may be linked to the global changes in epigenetic patterns.

Previous studies on insecticide resistance and the response to *P. falciparum* have suggested that insecticide resistant mosquitoes, regardless of whether it is mediated by target site or metabolic resistance, have reduced sporozoite prevalence [55], [56]. SENN DDT has been demonstrated to have both resistance mechanisms [32], and this may be related to the decreased *Def-1* expression and the decreased *Gamb* expression in response to Gram-positive bacterial challenge in the SENN DDT strain. However, there was not a marked difference in the expression of either *Gamb* of *Def* at the constitutive level or after Gram-negative treatment. The notable difference is the significantly increased Gamb expression in SENN-DDT in response to Gram-positive stimulation. Both of these peptides have been demonstrated to be involved in anti-*Plasmodium* defence [36], [57]. Defensin is active against early-stage *Plasmodium falciparum* gametocytes [58]. Gambicin is effective against the ookinete stage of *P. berghei* [36]. The effect of the insecticide resistant phenotype on vector competence cannot be clearly defined. However, there have been several examples of increased parasite prevalence and intensity in mosquitoes with both target-site and metabolic resistance. (as reviewed in [59], [60]. It is therefore interesting that SENN-DDT, which displays both target site and metabolic resistance, does not differ significantly in AMP expression SENN. This suggests that if differences in immune responsiveness does exist between the two strains, that it is not expressed at the level of AMP induction. There is a suite of other immune responses where these differences could exist. This includes activation of the pro-phenoloxidase (PPO) and complement cascades. However, it does display significantly lower levels of GNBP after Gram-positive challenge. There may still be further differences in immune active molecule expression that may alter *Plasmodium* permissiveness between the two strains that is not reflected in this experimental methodology.

GNBP, unlike the AMPs, is an inducer of the immune response rather than a consequence of immune induction. The lack of differences both constitutively and in response to Gram-negative stimuli therefore suggests a lack of species-specific differences in the induction of the Toll pathway. It is worth noting, however, that Gram-positive stimulation had a marked effect that differed from the patterns observed with the AMPs, with SENN being the highest, SANGWE the lowest and SENN and MAFUS in between. This does suggest that the potential for examining inducibility differences between the species as a potential source of interspecies immune variability. Indeed, *An. quadriannulatus* stimulate the PPO cascade at lower levels than *An. gambiae* [27]. This suggests that differences that may exist could be upstream of AMP production.

It must also be mentioned that it is unusual not to observe high AMP activity in the *An. quadriannulatus* strain SANGWE, for the reasons outlined above. The immune responsiveness of this non-vector may therefore be a result of early triggering of the response rather than by upregulation of AMP expression. However, this may an artefact of using bacteria rather than parasites as the stimuli. The responsiveness of *An. quadriannulatus* may be parasite-specific rather than general immunological stimuli.

A consistent pattern in the expression of AMPs is the difference in transcript levels between major vectors and minor/non-vectors. Typically, AMP transcript levels in MAFUS and SANGWE were higher than that of the *An. arabiensis* strains, even at the basal level. MAFUS always had higher AMP transcript levels than SENN. This is true for *Def-1* expression in SANGWE as well. However, there are notable exceptions of *Gamb* expression in SANGWE relative to SENN post Gram-negative bacterial exposure (no difference) and Gram-positive bacterial exposure (reduced). This suggests that AMP response to immune challenge may be a key factor underlying vector competence. These findings support previous findings of lower levels of induction of the immune response in *An. quadriannulatus* to *P. falciparum* [27].

Although the AMP expression patterns offer a potential correlation with vector competence, it is worth noting the consistently higher AMP transcript levels in MAFUS, despite it being a minor vector. Therefore, if the current hypothesis is correct, the AMP expression in MAFUS should be lower than that of the non-vector SANGWE. Contradiction to this hypothesis in this study could potentially be explained by the fact that MAFUS is reared in a concentration of 50 % salt water, as *An. merus* breeds in salt-water [61]. As several AMPs, including the Defensins CADEF1 and PDF1.2 have been demonstrated to be involved in osmotolerance (reviewed by [62]). It does also raise the question of larval salinity exposure and the immune response in *An. merus*.

Although there is a marked difference in AMP expression between strains, there are several considerations that must be taken. This study examined only mRNA levels, and as such may not be representative of the actual circulating peptides. AMPs are often post-translationally modified [63] and this could alter the *in vivo* functionality. Regardless, this marked difference must be considered and does support a hypothesis of variable immune responsiveness with the *An. gambiae* complex. This must be confirmed in field derived specimens, though.

There are several considerations in this study. The first are the limitations of this study. The first is that AMPs are only a single metric of the immune function. To fully implicate variable immune responses in differential vector competence in the *An. gambiae* complex, a range of humoral and cellular immune factors would have to be examined. AMPs were chosen for the simplicity of analysis. Secondly, this is a preliminary study as there very little work done on the epigenetics of the *An. gambiae* complex outside of the nominal members. The basic calorimetric tests could only examine global epigenetic changes and could not specifically link these changes definitely to gene silencing or activation. Although no mechanistic explanations are provided, this study sets the framework for potential studies examining an epigenetic basis for vector competence. The techniques used in this study has been demonstrated to be capable of detecting epigenetic changes in mosquitoes previously [64]. As the techniques have been demonstrated to be able to detect methylation and HAT activity shifts, the fact that constitutive interspecies differences can be detected does represent an actual change. However, these differences cannot only be attributed to their evolutionary distances as there is a mixture of differences in methylation as well as no significant differences. Due to this variability, the results are likely to be a result of the immune modulation rather than simply evolutionary distances. It must be stated, though, that this is a preliminary study and there are many more studies that must be performed. Next generation sequencing can be used to further probe these findings [65]. Finally, it must be noted that the ingested bacteria were not quantified post feeding. As such, differences may be due in part to variable amounts of bacteria ingested. However, due to the consistency of the results found across multiple feeds suggests that this is not the sole reason.

In conclusion, this study suggests a molecular underpinning for differences in the immune response of the major, minor and non-vectors. There is constitutive difference between major and minor vectors at the 5mC and m6A methylation levels, with Gram-positive and Gram-negative bacterial challenge resulting in a substantial change in the epigenetic landscape. The marked increase in AMP production in minor and non-vectors, particularly post immune stimulation suggests that this may be a major driver of immunological differences that underlie vector competence.

## Funding

This work was supported by the National Research Foundation of South Africa: Competitive Support for Unrated Researchers grant [grant numbers SRUG190313423259]; 10.13039/100000865Bill and Melinda Gates Foundation grant [Grant No. OPP1210314] and partly funded by the International Atomic Energy Agency (10.13039/501100004493IAEA) under their Technical Cooperation Programme [SAF 5014/5017] and NRF grants [Grant numbers 119765 and 107428].

## CRediT authorship contribution statement

**Nashrin F. Patel:** Writing – original draft, Investigation, Formal analysis, Data curation. **Blaženka D. Letinić:** Writing – review & editing, Methodology, Investigation, Formal analysis. **Dumisani M. Dlamini:** Resources, Investigation. **Nondumiso Mabaso:** Resources, Investigation. **Leanne N. Lobb:** Writing – review & editing, Validation, Project administration. **Jacek Zawada:** Writing – review & editing, Investigation, Formal analysis. **Givemore Munhenga:** Writing – review & editing, Supervision, Funding acquisition. **Shune V. Oliver:** Writing – review & editing, Supervision, Funding acquisition, Formal analysis, Conceptualization.

## Declaration of Competing Interest

The authors declare that they have no known competing financial interests or personal relationships that could have appeared to influence the work reported in this paper.
